# Veterinary Hygiene and the New York City Stables

**Published:** 1881-04

**Authors:** Allan S. Heath

**Affiliations:** Professor of Cattle, Sheep, and Swine Practice, Columbia Veterinary College and School of Comparative Medicine


### ART. X.—VETERINARY HYGIENE, AND THE NEW YORK CITY STABLES.

### No. II.

THE confinement of city horses and cows is so unnatural and severe, that, with the best sanitary conditions, their lives must be far short of those having more liberty. The labor lives of road horses in the city reaches about six and a half years; livery horses, six years; truck horses, owned by drivers, eight years; stage horses last seven years; express horses, seven and a half years; car horses, four and a half years only; while farm horses, driven by the owners, average twelve years of labor life.

Every horse requires 1,062 cubic feet of pure air every day to maintain perfect health; but in bad stables the air is vitiated, and the horse breathes in these poisons ten times every minute, with a rapid and certain vitiation of his blood, which completes its circuit every twenty seconds, thus shortening his life at every respiration. If man requires 80 cubic feet of air space, the horse requires at least 5,000 cubic feet.

An important factor in the life of the city horse is the temperature in the stable; for at rest he has two degrees less temperature than at liberty. He will suffer less at rest in a temperature too high, with plenty of pure air, than in a temperature too low; for the required temperature must be secured at the expense of food and air.

It is extremely rare for a city horse to breathe pure air in the stable, and he suffers in common with *human citizens* from vitiated and poisonous air in the streets. At his daily toil the horse breathes rapidly, and all of his physical functions are stimulated to the highest degree. His blood is rapidly poisoned by the foul air of the city streets, and by disease germs floating in every dust-cloud that daily sweeps through the city in dry weather.

After a day of severe and exhausting toil, the city horse is brought into a cold, dark, damp stable, having a wooden floor, reeking in filth, and emitting foul odors; he is fed on poor, musty hay and hard, dry grain, and too often of bad quality, and his thoughtless owner wonders when he finds his faithful horse unfit for six days' toil a week, and a long and rapid drive on Sunday.

In the vile stables of New York, Mr. Henry Bergh can find more *cruelty to animals* than is ever exhibited in the streets in broad daylight by the most depraved wretches who abuse dumb brutes.

Wood is unfit for stable floors, because of its drying and heating nature to the delicate textures of the horse's feet, and its capability to absorb and retain offensive and decomposing matters in the urine.

Brick laid on edge on sand, and cemented in by melted asphalt, makes an impervious, cleanly, and cool flooring for stables, which can be kept free from ammoniacal odor and decomposition by being daily sprinkled with a few pounds of ground plaster—sulphate of lime—and littered at night with clean straw. The stables thus treated remain sweet, and the litter can be used twice the period of that handled in the usual way. It is essentially important to care for the horse's feet; for they are the first and chief seat of disease, and lameness the most common cause of injury and impaired usefulness.

Cleanliness in the stables, and frequent removal of manure, and disinfection of the manure vaults, prevent flies from tormenting stabled horses in warm weather. The *stomoxys calcitrans* lays its eggs in the warm, fermenting manure, and hatches in large numbers. This fly is a tormenting blood-sucker, and greatly annoys horses, and especially such as a bad fashion has deprived of its natural defence, by cutting the long flowing tail into a short stub, utterly powerless to drive flies from the defenceless brute. This insect, in the pupal state, is the common manure maggot.

Lime and carbolic acid destroy both the eggs and the maggots; but cleanliness is the preventive measure of greatest value.

Flies are driven out of stables by hanging up bunches of plantain leaves previously dipped in milk. A decoction of ergot sprinkled about the stables will also destroy flies. Thin-skinned horses suffer intensely, losing in condition by the painful stings of flies. This may be prevented by moistening the horse-brush in a decoction of walnut-leaves, and slightly moistening the coat after the morning grooming. Washing the horse's legs and feet in a solution of borax will prevent the annoyance from flies. But the full tail is a horse's natural defence against them. The man who bangs his horse's tail in the summer is a fit subject for prosecution by the Society for the Prevention of Cruelty to Animals.

City cow stables are generally filthy; their management is filthy; the people are filthy; the pails, pans, cans, strainers, &c., &c., are extremely filthy. Filth is the soil for the growth and incubation of disease germs. In the unbuilt-up lots and breathing-places for citizens are located the city cow stables, contaminating and vitiating the atmosphere for blocks in every direction. The wind may be fickle in bearing odors in excess at times; but the stench from city cow stables is never-failing, is disgustingly constant, spreading disease and death to those who seek pure air in the suburbs.

From the Battery to Harlem river, and from the Hudson to East river, there is now little enough pure air for human beings to breathe, without dividing it, and poisoning it with useless animals, emitting offensive and baneful odors. Mechanics must yet achieve the application of unobjectionable motor power, which shall run our street cars, move our merchandize, and handle our commodities with the least possible aid from horses. Such appliances would add vastly to the sanitary condition of New York, and other large and crowded cities. All horse stables should possess every sanitary arrangement; and cow stables are unnecessary and unwarrantable nuisances, that should be abated by Boards of Health.

City stables should be built in special localities, where, congregated together, every sanitary provision may be made to secure cleanliness and freedom from every kind of offence; and where repeated inspection shall secure and maintain the health of the city. In this way the animals stabled in them may be kept in a healthy condition, and the proximity of stables to residences may, and should be, prohibited.

The sanitary condition of the stabling of 7,000,000 of horses, and the 4,000,000 of cows in the State of New York, would add many millions of dollars to the value of these properties in improvement and saving. Could this benefit extend to the care and protection of the 600,000,000 of horses, and to the 300,000,000 of cows, and also to the 1,000,000,000 of cattle in the United States, it would raise the price of cattle from the low average of $20 per head to at least $25 or $30; and would raise the average price of horses from $58 to $75 per head, making an enormous grand total of added wealth to the country.

Perfect sanitary city stables would secure a marvellous decline of the mortality of our citizens from nearly two hundred per day, to the lowest death rate of the most civilized city of Europe.

The number of city-kept cows is decreasing in New York year by year, though it is difficult to secure pure milk for our two millions of citizens and visitors from the best rural sections within a radius of one hundred miles. It is certainly more than doubtful propriety to attempt to produce milk, within the densely populated city, from city-fed and city-stabled cows. There is an error in the minds of many citizens, that milk is pure and good as it comes from the cow near by, and unwatered—always from the same cow. The fact is, that good material, in the shape of sound food, pure water, exercise, light, air, and all sanitary surroundings, are the important elements in the production of sweet, healthy, and sound milk. The milk of city-stabled cows is more or less dangerous, from the fact that it often contains tuberculous matter from the phthisis in the cow, produced by unsanitary conditions. Feeble young children consuming tuberculous milk, suffer from diarrhœa; and if long fed upon it, die of consumption. Tuberculosis in the city cow is common, as the result of confinement in dark, damp, filthy stables, and of its being fed on bad food.

ALLEN S. HEATH, M.D.,
*Professor of Cattle, Sheep, and Swine Practice,*
*Columbia Veterinary College*
*and School of Comparative Medicine.*

